# Use of Minimally Invasive Surgery in the Diagnosis and Treatment of Cancer in Dogs and Cats

**DOI:** 10.3390/vetsci6010033

**Published:** 2019-03-20

**Authors:** Ingrid M. Balsa, William T. N. Culp

**Affiliations:** Department of Surgical and Radiological Sciences, University of California-Davis, 1 Shields Ave., Davis, CA 95616, USA; wculp@ucdavis.edu

**Keywords:** Surgical oncology, neoplasia, minimally invasive surgery, staging, thoracoscopy, laparoscopy

## Abstract

Surgical management of neoplastic disease is common in veterinary medicine. Minimally invasive surgery (MIS) has gained widespread acceptance by veterinary surgeons and is experiencing rapid growth and frequency of use. Many neoplastic diseases in the abdomen and thorax of dogs and cats can be treated as effectively with MIS as with traditional open surgery. Additionally, MIS allows for less invasive options for organ biopsy in cancer patients either for initial diagnosis or for staging to inform prognosis and treatment. Despite the recent increase in MIS, additional research is required to further characterize the benefits to oncology patients and to ensure that surgical oncologic principles and patient outcomes are not compromised by the use of MIS.

## 1. Introduction

Despite the regular use of minimally invasive surgery (MIS)—specifically laparoscopy and thoracoscopy—in human practice for decades, acceptance of MIS in veterinary medicine as standard-of-care has occurred more slowly. This is likely multi-factorial and may be associated with the cost of instrumentation, size of patients and the learning curve of new surgical skills. Initial descriptions of veterinary diagnostic laparoscopy were published in 1977 [1]; however, it was not until year 2000 that there was widespread acceptance and interest among veterinary surgeons. Since that time there has been exponential growth in the field for the management of both benign and neoplastic conditions. Likewise, the discipline of veterinary surgical oncology has grown considerably as a discipline since the mid-1980s when specific surgical oncology fellowship training was made available to veterinary surgeons. In oncology patients, the use of MIS is considered desirable due to the potential for decreased pain, more rapid return to function and potential decreased inflammation following surgery [2,3].

Throughout the veterinary MIS literature, the importance of case selection is evident. When choosing MIS oncology candidates, considerations must include the size of the tumor, size and body shape of the patient (e.g., Labrador retriever versus English bulldog), potential presence of adhesions and the patient’s ability to tolerate a pneumoperitoneum if pulmonary, cardiovascular or intracranial comorbidities exist. 

A wide variety of available instruments are desirable in veterinary MIS due to the variable size of patients. Port cannulas generally range from 2–12 mm with 5- and 10-mm instruments being most commonly used. Vessel sealing devices and stapling equipment are regularly used and necessary for many procedures. Due to the cost of equipment, re-sterilization and reuse of instruments that are intended for single use (e.g., LigaSure^TM^ handpiece and SILS^TM^ port) is common in veterinary medicine. There is growing literature to support the safety of this practice [4,5]. Step-by-step descriptions of surgical procedures are outside the scope of this manuscript; the authors suggest further reading in textbooks for more in-depth discussions regarding surgical technique prior to implementing MIS in a veterinary practice [6]. Additionally, the tumor size guidelines presented in this manuscript are meant to characterize the knowledge in the current veterinary literature and should not be taken as absolute recommendations and will vary from patient to patient. Also, as surgeon skill and sophistication of surgical instruments increase, larger tumors in smaller patients may well be able to be safely removed utilizing MIS. 

## 2. Laparoscopy

### 2.1. Splenectomy

The canine spleen is a significantly larger organ relative to body size than the human spleen. Therefore, although laparoscopic splenectomy has gained widespread acceptance in human medicine for hematologic conditions and trauma, it is less commonly performed in veterinary patients. The most common indication for canine splenectomy is presence of a splenic mass. Splenic size combined with the addition of a mass may be the reason why laparoscopic splenectomy in veterinary patients has been limited. However, both multi-port and single-incision techniques have been described as well as total laparoscopic and laparoscopic-assisted techniques. At the time of this writing, 69 laparoscopic or laparoscopic-assisted canine splenectomies have been described in the literature [7,8,9,10,11]. The most common reason for having this procedure performed is the presence of a splenic mass. Splenic masses larger than 6 cm are considered a relative contraindication to MIS as visualization may be impaired by sheer size and omental adhesion; additionally, the ability to manipulate the spleen may be decreased potentially leading to a greater chance of splenic rupture. Patient selection is also generally limited to dogs without a hemoperitoneum due to concerns about visualization. 

The most common malignant tumors of the canine spleen are hemangiosarcoma, histiocytic sarcoma and lymphoma [12]. For these diseases, treatment generally includes splenectomy and adjuvant chemotherapy. Laparoscopic splenectomy has been described successfully in 3 cats in the veterinary literature; management of mast cell disease appears to be a common reason to consider this procedure in cats [13].

At the time of this writing it is unknown if MIS splenectomy alters the outcome of these patients compared to patients that undergo a splenectomy via open celiotomy. Conversion to an open procedure may be required in cases of hemorrhage. While this is reported in the human literature it has not been reported as a reason for conversion in the veterinary literature, likely due to case selection and though not reported the possibility of ongoing hemorrhage requiring conversion exists in veterinary patients. The only conversion during a laparoscopic splenectomy reported in the veterinary literature was due to severe omental adhesions and abundant falciform fat obscuring the surgical field [9]. Post-operative complications include surgical site bruising, infection and inflammation which may be decreased in dogs undergoing MIS splenectomy as compared to an open splenectomy although prospective studies to evaluate this further are needed [9,14].

Proposed benefits of laparoscopic splenectomy include decreased pain following surgery and decreased rate of infection [14]. Generally short-term outcomes following laparoscopic or laparoscopic-assisted splenectomy are considered excellent with all reported cases surviving to discharge. Long-term outcome in dogs with malignant splenic tumors is guarded; however, this outcome is likely not influenced by the type of splenectomy (MIS vs open) the dog undergoes. 

### 2.2. Adrenalectomy

Laparoscopic adrenalectomy is a common procedure in both human and veterinary medicine for non-invasive adrenal tumors of small to moderate size. In both human and veterinary patients multi-port and single-incision laparoscopy has been described; additionally, both transperitoneal and retroperitoneal approaches have been described in humans and dogs [15,16]. In dogs in which an adrenal tumor has been diagnosed, the first step is a complete endocrine evaluation to determine if the tumor is functional or non-functional. It is important to remember that both adenomas and carcinomas may be either functional or non-functional, and therefore, despite their benign nature many veterinary patients diagnosed with adrenal gland adenomas are still reasonable surgical candidates [17]. Currently, approximately 50 clinical laparoscopic adrenalectomy surgeries in dogs and 12 in cats are described in the veterinary literature [16,17,18,19,20]. 

Adrenal cortical carcinoma is an aggressive and highly metastatic tumor in humans, with over 20% of patients having nodal or distant metastasis at the time of diagnosis [21]. This is contrary to canine adrenal cortical carcinoma where less than 14% have evidence of metastasis at the time of diagnosis [22,23]. In human medicine there are some studies to support that laparoscopic adrenalectomy may be inferior to open adrenalectomy based on recurrence and disease-free interval, the long-term outcomes in veterinary patients is not yet known [24,25,26]. In dogs with functional cortical carcinomas the most common hormone produced is cortisol, resulting in hyperadrenocorticism. Clinical signs of hyperadrenocorticism are important differentiators between functional and non-functional tumors. Diagnosis is via an elevated urine cortisol:creatinine ratio and either ACTH stimulation or low-dose dexamethasone suppression test. In cats, the most common adrenal tumor is an aldosterone-secreting tumor which is diagnosed via a plasma aldosterone level.

Pheochromocytoma or a tumor of the chromaffin cells of the adrenal medulla, secretes catecholamines. These tumors are uncommon in dogs and rare in cats. Urine normetanephrine:creatinine ratio is useful in differentiating catecholamine secreting tumor and non-functional tumors [27]. Metastasis at diagnosis in dogs is approximately 20% and reported common locations include the regional lymph nodes, lung, liver, spleen and kidney [28,29].

Vascular invasion is considered a contraindication to laparoscopic adrenalectomy and this is encountered more commonly with malignant tumors (e.g., pheochromocytomas and adenocarcinomas) than adenomas [22,30,31]. Therefore, a contrast computed tomography (CT) angiogram is considered mandatory as a pre-operative diagnostic by the authors. Relative contraindications to a laparoscopic technique include tumor size; tumors > 7 cm are generally approached via an open celiotomy, but this varies somewhat based on the size of the patient and surgeon experience [32]. Additionally, it is thought that left-sided and cranial pole tumors are easier to resect than right-sided or caudal pole tumors due to the intimate association of the right adrenal gland with the vena cava and the caudal pole of the adrenal gland with the renal vasculature. Complications of laparoscopic adrenalectomy include hemorrhage, which can be life-threatening and require emergent conversion to an open celiotomy and capsular penetration. The consequence of capsular penetration in veterinary patients is unknown at this time. Intraoperative complications altering anesthesia during resection of pheochromocytomas can be attributed to catecholamine release and include changes in blood pressure and cardiac arrhythmias. To minimize risk of death, pretreatment with phenoxybenzamine has been advocated in open surgery prior to pheochromocytoma resection [33]. Pancreatitis and thromboembolic complications are well-documented complications following adrenalectomy and these complications may be decreased with laparoscopic adrenalectomy compared with open adrenalectomy [17].

### 2.3. Liver Biopsy

Laparoscopic liver biopsy is a commonly performed procedure for diagnosing a variety of hepatobiliary diseases in both veterinary and human patients. Hundreds of canine laparoscopic liver biopsy cases (fewer feline) have been reported in the literature owing to the relative ease of the procedure and the frequency with which it is performed. The efficacy of laparoscopic liver biopsy has been shown to be similar to that of biopsy obtained by open celiotomy and superior to that obtained by percutaneous cutting needle [34]. 

Laparoscopic liver biopsies have been described using either a multi-port technique or a single-incision port. Historical contraindications to laparoscopic liver biopsy include thrombocytopenia (<80,000/dL), increased prothrombin time and/or partial thromboplastin time and ascites. However, a recent large retrospective study on dogs undergoing laparoscopic liver biopsies did not find any difference in outcome in dogs with those contraindications. Additionally, all samples were of sufficient size for a histopathologic diagnosis [35]. Therefore, these contraindications should be considered on a patient-by-patient basis, taking into consideration the resources of the hospital and accessibility of blood products if needed. Liver biopsy of solitary masses may result in substantial hemorrhage and has been shown to be a risk factor for conversion during diagnostic laparoscopic procedures [36]. 

The most commonly reported complication of laparoscopic liver biopsy is hemorrhage from the biopsy site or from splenic laceration during port placement, and the need for conversion to an open procedure has been documented in 2–7% of dogs [35,37]. Other complications include port site infection or inflammation. Short-term outcomes of laparoscopic liver biopsy are excellent, with >95% of patients surviving to discharge; non-survival is more frequently due to progression of the underlying disease and not from complications arising from laparoscopic liver biopsy. 

### 2.4. Renal Biopsy/Nephrectomy 

Laparoscopic renal biopsy is performed in veterinary medicine for a variety of diseases including neoplasia but is more commonly performed for dogs and cats experiencing proteinuria, unexplained renomegaly and infiltrative renal disease [38]. Common renal tumors in dogs include renal cell carcinoma, transitional cell carcinoma and metastatic lesions. In cats the most common renal neoplasia is lymphoma. Alternatives to laparoscopy for renal biopsies exist, including ultrasound-guided needle biopsies; however one study demonstrated improved sample quality with laparoscopic-acquired biopsy as compared to ultrasound-guided biopsy [39]. Benefits of laparoscopy over ultrasound may also include control of hemorrhage at the biopsy site due to the ability to apply direct pressure to the site and direct visualization of the lesion. Laparoscopic renal biopsy has been described using a single or multi-port technique and ideally a 16-gauge spring-loaded automated biopsy needle [40].

Contraindications for renal biopsy are severe anemia, stage 4 chronic kidney disease, coagulopathy, hypertension, hydronephrosis, pyelonephritis and cystic renal disease. Complications associated with renal biopsy include non-diagnostic samples, hemorrhage, hematuria, perirenal hematoma, hydronephrosis, infarction, ureteral laceration, urinary obstruction secondary to blood clot formation and decreased renal function; complication rates range from 1–20% [38,41].

Laparoscopic ureteronephrectomy is another uncommon surgery that may be performed in cases of unilateral renal neoplasia in dogs and cats. This surgery, combined with removal of regional lymph nodes and surrounding tissue, including potentially the ipsilateral adrenal gland, is commonly performed in human patients with renal cell carcinoma [42,43]. Within the human literature, dogs have been used as an experimental model of laparoscopic ureteronephrectomy and have been shown to have decreased surgical stress compared with those undergoing open surgeries [44]. Surgical descriptions in dogs include multi-port techniques and case selection for neoplasia has been limited to 7 cm unilateral lesions with very large tumors being considered a contraindication to laparoscopic surgery [45]. Both a renal cystadenocarcinoma and an infiltrative epithelial neoplasm have been laparoscopically removed in dogs. Contraindications other than tumor size may include severe hydronephrosis/hydroureter or severe pyelonephritis or infection extending beyond the renal capsule. 

Conversion rate of laparoscopic ureteronephrectomy in the literature is 22% which suggests this is a technically challenging surgery [45]. Other complications include hemorrhage and port site infection. Reports of port site metastasis following removal of renal cell carcinoma exist in the human literature, however, this has not yet been reported in veterinary medicine [46].

### 2.5. Ovariohysterectomy/Cryptorchidectomy

Laparoscopic ovariohysterectomy and cryptorchidectomy are commonly performed as elective sterilization surgeries in veterinary medicine. Therefore, reproductive tumors in the dog and cat are much less common than they are in human medicine. Regardless, laparoscopy has been used for removal of tumors of the female reproductive system. There is a single case report of laparoscopic removal of a 7 cm ovarian teratoma via a laparoscopic-assisted ovariohysterectomy [47]. Although not reported in the veterinary literature, it is also reasonable to consider that laparoscopy could be used to excise small to moderately sized uterine tumors. 

Cryptorchidism is a congenital abnormality in dogs that may be unilateral or bilateral. For testicles retained with in the abdomen neoplastic conversion is 13.6 times more likely compared with testicles in the scrotum of dogs [48]. Laparoscopic cryptorchidectomy has been reported with both multi-port and single incision techniques. Laparoscopic cryptorchidectomy has been utilized for the removal of a seminoma, interstitial cell tumor and testicular mixed cell tumor in dogs [49]. Prognosis for all of these tumor types is excellent with complete excision. Due to the congenital nature of cryptorchidism, both testicles should always be removed. 

Due to the lack of numbers, no definitive case selection criteria for reproductive neoplasia requiring ovariohysterectomy or cryptorchidectomy is available. However, size of tumor relative to size of dog should be taken into account as well as ability to obtain acceptable surgical margins, as surgical oncologic principles should not be sacrificed for the sake of performing a minimally invasive procedure.

## 3. Thoracoscopy

### 3.1. Lung Lobectomy

Lung lobectomy both via video-assisted thoracoscopic surgery (VATS) and by a totally thoracoscopic technique has been reported in dogs for the removal of primary lung tumors and metastatic pulmonary lesions [50,51,52]. These techniques are most appropriate for smaller masses that are located at the periphery of the lung lobe [50,51,52]. The minimally invasive technique described for lung lobectomy has utilized multiple ports. Successful thoracoscopy also often requires placement of bronchial blockers for one-lung ventilation (OLV) and an understanding of the physiologic consequences that accompany this procedure. One-lung ventilation is considered important because it allows for better visualization of the mass and the hilus of the lung and increases the working space within the chest [53]. Currently in the veterinary literature thoracoscopic lung lobectomy has been reported in 22 dogs with neoplasia, with carcinoma being diagnosed most commonly followed by histiocytic sarcoma [50,51]. This is similar to previous reports in which adenocarcinomas were found to be the most common lung tumors in dogs [54]. Lung tumors and metastatic lesions up to 7 cm in size have been removed thoracoscopically [50]. Currently in the veterinary literature VATS lung lobectomy has been reported in over 30 dogs with tumor volumes up to 174.4 cm^3^ being successfully resected [52,55,56,57]. 

Contraindications to thoracoscopy in companion animals have not been fully elucidated, however in humans, these include diffuse body wall neoplasia or pleural adhesions and respiratory compromise with subsequent inability to tolerate OLV. With regards to tumor characteristics, very large tumors or those close to the hilus have been considered poor candidates for MIS [53]. Due to the inability to perform a thorough thoracic exploration, the authors believe that a thoracic CT scan is a mandatory pre-operative diagnostic to identify metastatic disease either within the lung lobe being treated, in other lung lobes or within regional lymph nodes. Complications include the need for conversion to an open thoracotomy, with reported conversion rate estimates ranging from 23–44% [50,51]. Reasons for conversion include hemorrhage (intercostal artery), failure to maintain OLV and poor access/visualization due to location of the tumor [50,51]. This is slightly higher than conversion rates in the human literature (18%) for VATS resection of primary lung tumors [58]. Other complications include port site inflammation and infection; infection in these cases can extend into the thoracic cavity and result in pyothorax. There has been no significant differences identified between dogs that had lung tumors removed via thoracoscopy or VATS or via open thoracotomy [50,52]. This is similar to the current human literature in which both morbidity and mortality outcomes appear to be similar for MIS versus open thoracotomy [58,59]. 

MIS lung biopsies have been described for diffuse pulmonary disease as well as small peripheral neoplastic masses. Loop ligatures, stapling devices and vessel sealing devices have all been used for peripheral lung biopsies [60,61]. 

### 3.2. Mediastinal Masses

Thoracoscopic removal of mediastinal masses has been reported in both the dog and the cat [62]. The most common mediastinal mass in the dog is thymoma followed by lymphoma. The most common mediastinal mass in the cat is lymphoma and therefore, mediastinal masses are less commonly surgically removed in cats. Other potential neoplastic mediastinal masses include carcinoma, most commonly of thyroid origin and hemangiosarcoma, among others [62]. In the 18 dogs reported in one study, the largest tumor removed thoracoscopically had a tumor volume of 313.3 cm^3^ [62]. 

Complications associated with mediastinal mass excision include the need for conversion which occurred in 11% of dogs in one study [62]. Another interesting complication is port site metastasis which occurred in one dog 6.5 months following thoracoscopic thymoma removal [63] and in one dog following thoracoscopic pleural and mediastinal biopsy [64]. Port site metastasis is more commonly reported in human medicine (~1% of MIS cases) than in veterinary medicine potentially due to the life span of the patient. The cause of port site metastasis is incompletely understood but likely includes level of patient debilitation, presence of disseminated disease, contamination of the cannulas with highly exfoliative tumor cells or contamination of the portal incisions during unprotected tumor removal. Additionally, the ‘chimney effect’ due to the expulsion of insufflation gas droplets at portal sites is suspected in cases where insufflation is utilized. Neither of the two case reports of thoracoscopic port site metastasis involved insufflation. In one dog, a large amount of pleural effusion existed and this may have played a role in the development of the metastasis [64]. 

### 3.3. Right Auricular Mass Resection and Pericardiectomy

Thoracoscopic right auricular mass resection has been described in 10 dogs in the veterinary literature with good short-term outcome [65,66]. Multi-port techniques have been described with lesions up to 2 cm in size located at the apex of the right auricle. Short-term outcomes appear good with this technique with 9 of 10 dogs surviving surgery, however this is likely highly dependent on surgeon skill. Long-term outcome is guarded as 9 of the 10 lesions were consistent with hemangiosarcoma and those dogs had a mean survival time of 90 days (range, 0 to 251 days) [65]. This is similar to survival time reported for dogs undergoing right auricular hemangiosarcoma resection via open thoracotomy when followed by adjuvant chemotherapy [67]. 

The major potential complication with thoracoscopic right auricular mass removal is hemorrhage, which will quickly become life threatening if there is rupture of the right atrium or failure of the surgical staple line. Additional complications include port site infection or inflammation which is particularly important in these patients as it can delay initiation of systemic chemotherapy. 

A pericardial window is performed prior to right auricular mass resection, with the purposes of relieving cardiac tamponade and allowing full evaluation of the size and location of the mass via pericardioscopy. If the mass lesion is large or approaching the base of the auricle, conversion to an open thoracotomy should be pursued [68]; if the mass is not surgically resectable the pericardial window may be palliative in the short-term. A pericardial window or subphrenic pericardiectomy may prolong survival time in dogs with aortic body tumors [69]; this same advantage has not been appreciated in dogs diagnosed with right auricular hemangiosarcoma if the primary mass is not removed at the time of surgery [70]. 

A multi-port thoracoscopic technique has been described with patients positioned in both dorsal and lateral recumbency. Determining the amount of pericardium to remove is controversial with current recommendations on the side of “more is better” in cases of idiopathic pericardial effusion [71,72]. This is likely less of a concern in cases with effusion secondary to neoplasia due to the overall shorter expected survival times in this patient population. In one study, removal of a 4 × 5 cm piece of pericardium was considered sufficient for palliation of clinical signs [73]. If a definitive diagnosis has not been obtained prior to surgery, a subphrenic pericardiectomy is preferable. A novel technique of pericardial window with vertical pericardial fillets has been reported in dogs but not evaluated for neoplastic pericardial effusions [74]. 

Contraindications to performing a thoracoscopic pericardiectomy may include patients that are unable to undergo OLV due to cardiac or respiratory disease. However, a subphrenic pericardiectomy can be performed safely in a dog without OLV [75]. Breed conformation is likely a major factor with regards to working space with barrel-chested dogs having limited space compared with deep-chested dogs. This lack of space is exacerbated in cases when the pericardium is distended with fluid. Therefore, pre-operative pericardiocentesis is advised prior to thoracoscopy; additionally, fluid drainage will improve cardiovascular stability during anesthesia [76]. Complications specific to thoracoscopic pericardiectomy include fatal ventricular arrhythmias, thought to be secondary to use of an electrocautery device, hemorrhage and damage to the phrenic nerve.

## 4. Lymph Node Staging

Knowledge of lymph node metastasis in cancer patients is important staging information with tumor, node, metastasis (TNM) systems described for several tumor types. Lymph node metastasis is often determined via ultrasound-guided fine needle aspiration and cytology. However, this modality may be difficult for certain lymph nodes especially if small or near vital structures. Laparoscopic lymphadenectomy allows for the added benefits of direct visualization, potentially minimizing damage to surrounding tissue and the acquisition of larger tissue samples for histopathology. 

Options for lymph node removal include both locoregional lymph nodes and sentinel lymph nodes. Historically locoregional lymph nodes have been removed in veterinary patients if they are grossly enlarged, known to be metastatic or if easily accessible through the primary incision site. This technique may under-stage patients due to lack of detection of micrometastasis in lymph nodes of normal size. Additionally, while it seems reasonable that locoregional lymph nodes would be the most likely to contain metastasis, sentinel lymph node mapping in veterinary medicine has shown that the regional lymph node is not always the sentinel lymph node [77,78]. The sentinel lymph node is defined as the first node to receive afferent lymphatic drainage from the tumor and can be variable. Therefore, if the sentinel lymph node is free of metastasis, it is reasonable to consider other lymph nodes free of metastasis. Sentinel lymph node mapping is widely used in human oncology patients, initially described for use in breast cancer and melanoma, however it is now used for many different tumor types and locations [79].

While many pre-operative imaging techniques for sentinel lymph node mapping have been described, intra-operative imaging including lymphoscintigraphy, blue dye techniques and near-infrared imaging dyes many be considered more useful to the surgeon especially when dissecting lymph nodes that are not enlarged as they may aid in visualization and differentiation from surrounding fat. Regardless of what technique is chosen a small amount of dye is injected peritumorally and within seconds to minutes uptake should be visualized within the lymphatics and eventually the sentinel lymph node [80,81].

The most common interface of sentinel lymph node mapping and MIS in veterinary medicine includes the iliosacral lymphatic center in dogs with apocrine gland anal sac adenocarcinoma in which nodal metastasis is common. A multi-port technique has been described in both experimental dogs with normal sized lymph nodes and clinical patients [82,83]. Sentinel lymph node mapping has also been employed during thoracoscopic removal of tracheobronchial lymph nodes in dogs and cats with pulmonary carcinomas [81,83]. 

Complications of MIS lymph node removal include rupture or tearing of the lymph node leading to regional seeding, and hemorrhage and damage to adjacent structures, including ureters, when dissection of medial iliac lymph nodes is being performed. 

## 5. Future Directions

Given its relatively recent adoption in veterinary medicine, MIS has proven useful for many aspects of veterinary surgical oncology. However, as is apparent in this review, case numbers are lacking for many techniques. In the few studies that do compare patient outcomes between MIS and open surgical oncology procedures there does not appear to be a difference in patient outcomes, and MIS may afford additional benefits such as patient comfort and faster return to function. More prospective studies are needed to fully understand the benefits of MIS in veterinary oncology patients. Given the additional costs and training associated with MIS techniques it is possible that it may not gain as widespread acceptance and practice in veterinary medicine as it has in human medicine.

MIS has opened the veterinary community to other palliative treatment options for cancer including MIS-assisted thermal ablation and the combination of MIS and interventional radiology techniques. As mentioned previously, much research is needed regarding use of these different modalities in veterinary medicine. 

## References

[1] Johnson G.F., Twedt D.C. (1977). Endoscopy and laparoscopy in the diagnosis and management of neoplasia in small animals. Vet. Clin. N. Am..

[2] Culp W.T., Mayhew P.D., Brown D.C. (2009). The effect of laparoscopic versus open ovariectomy on postsurgical activity in small dogs. Vet. Surg..

[3] Devitt C.M., Cox R.E., Hailey J.J. (2005). Duration, complications, stress, and pain of open ovariohysterectomy versus a simple method of laparoscopic-assisted ovariohysterectomy in dogs. J. Am. Vet. Med. Assoc..

[4] Coisman J.G., Case J.B., Clark N.D., Wellehan J.F., Ellison G.W. (2013). Efficacy of decontamination and sterilization of a single-use single-incision laparoscopic surgery port. Am. J. Vet. Res..

[5] Petrovsky B., Monnet E. (2018). Evaluation of efficacy of repeated decontamination and sterilization of single-incision laparoscopic surgery ports intended for 1-time use. Vet. Surg..

[6] Fransson B.A., Mayhew P.D. (2015). Small Animal Laparoscopy and Thoracoscopy.

[7] Mayhew P.D., Sutton J.S., Singh A., Runge J.J., Case J.B., Griffin M.A., Giuffrida M.A. (2018). Complications and short-term outcomes associated with single-port laparoscopic splenectomy in dogs. Vet. Surg..

[8] Wright T., Singh A., Mayhew P.D., Runge J.J., Brisson B.A., Oblak M.L., Case J.B. (2016). Laparoscopic-assisted splenectomy in dogs: 18 cases (2012–2014). J. Am. Vet. Med. Assoc..

[9] Shaver S.L., Mayhew P.D., Steffey M.A., Hunt G.B., Mayhew K.N., Culp W.T. (2015). Short-term outcome of multiple port laparoscopic splenectomy in 10 dogs. Vet. Surg..

[10] Collard F., Nadeau M.E., Carmel E.N. (2010). Laparoscopic splenectomy for treatment of splenic hemangiosarcoma in a dog. Vet. Surg..

[11] Khalaj A., Bakhtiari J., Niasari-Naslaji A. (2012). Comparison between single and three portal laparoscopic splenectomy in dogs. BMC Vet. Res..

[12] Cleveland M.J., Casale S. (2016). Incidence of malignancy and outcomes for dogs undergoing splenectomy for incidentally detected nonruptured splenic nodules or masses: 105 cases (2009–2013). J. Am. Vet. Med. Assoc..

[13] O’Donnell E., Mayhew P., Culp W., Mayhew K. (2013). Laparoscopic splenectomy: Operative technique and outcome in three cats. J. Feline Med. Surg..

[14] Stedile R., Beck C.A.C., Schiochet F., Ferreira M.P., Oliveira S.T., Martens F.B., Tessari J.P., Bernades S.B.L., Oliveira C.S., Santos A.P. (2009). Laparoscopic versus open splenectomy in dogs. Pesquisa Veterinária Brasileira.

[15] Ko J., Jeong J., Lee S., Son H., Kweon O.K., Kim W.H. (2018). Feasibility of single-port retroperitoneoscopic adrenalectomy in dogs. Vet. Surg..

[16] Jimenez Pelaez M., Bouvy B.M., Dupre G.P. (2008). Laparoscopic adrenalectomy for treatment of unilateral adrenocortical carcinomas: Technique, complications, and results in seven dogs. Vet. Surg..

[17] Mayhew P.D., Culp W.T., Hunt G.B., Steffey M.A., Mayhew K.N., Fuller M., Della-Maggiore A., Nelson R.W. (2014). Comparison of perioperative morbidity and mortality rates in dogs with noninvasive adrenocortical masses undergoing laparoscopic versus open adrenalectomy. J. Am. Vet. Med. Assoc..

[18] Pitt K.A., Mayhew P.D., Steffey M.A., Culp W.T., Fuller M.C., Della Maggiore A., Nelson R.W. (2016). Laparoscopic adrenalectomy for removal of unilateral noninvasive pheochromocytomas in 10 dogs. Vet. Surg..

[19] Mitchell J.W., Mayhew P.D., Culp W.T.N., Brad Case J., Singh A., Fuller M.C., Della Maggiore A. (2017). Outcome of laparoscopic adrenalectomy for resection of unilateral noninvasive adrenocortical tumors in 11 cats. Vet. Surg..

[20] Naan E.C., Kirpensteijn J., Dupre G.P., Galac S., Radlinsky M.G. (2013). Innovative approach to laparoscopic adrenalectomy for treatment of unilateral adrenal gland tumors in dogs. Vet. Surg..

[21] Bilimoria K.Y., Shen W.T., Elaraj D., Bentrem D.J., Winchester D.J., Kebebew E., Sturgeon C. (2008). Adrenocortical carcinoma in the United States. Cancer.

[22] Kyles A.E., Feldman E.C., De Cock H.E., Kass P.H., Mathews K.G., Hardie E.M., Nelson R.W., Ilkiw J.E., Gregory C.R. (2003). Surgical management of adrenal gland tumors with and without associated tumor thrombi in dogs: 40 cases (1994–2001). J. Am. Vet. Med. Assoc..

[23] Schwartz P., Kovak J.R., Koprowski A., Ludwig L.L., Monette S., Bergman P.J. (2008). Evaluation of prognostic factors in the surgical treatment of adrenal gland tumors in dogs: 41 cases (1999–2005). J. Am. Vet. Med. Assoc..

[24] Brix D., Allolio B., Fenske W., Agha A., Dralle H., Jurowich C., Langer P., Mussack T., Nies C., Riedmiller H. (2010). Laparoscopic versus open adrenalectomy for adrenocortical carcinoma: Surgical and oncologic outcome in 152 patients. Eur. Urol..

[25] Miller B.S., Ammori J.B., Gauger P.G., Broome J.T., Hammer G.D., Doherty G.M. (2010). Laparoscopic resection is inappropriate in patients with known or suspected adrenocortical carcinoma. World J. Surg..

[26] Miller B.S., Gauger P.G., Hammer G.D., Doherty G.M. (2012). Resection of adrenocortical carcinoma is less complete and local recurrence occurs sooner and more often after laparoscopic adrenalectomy than after open adrenalectomy. Surgery.

[27] Quante S., Boretti F.S., Kook P.H., Mueller C., Schellenberg S., Zini E., Sieber-Ruckstuhl N., Reusch C.E. (2010). Urinary catecholamine and metanephrine to creatinine ratios in dogs with hyperadrenocorticism or pheochromocytoma, and in healthy dogs. J. Vet. Intern. Med..

[28] Barthez P.Y., Marks S.L., Woo J., Feldman E.C., Matteucci M. (1997). Pheochromocytoma in dogs: 61 cases (1984–1995). J. Vet. Intern. Med..

[29] Gilson S.D., Withrow S.J., Wheeler S.L., Twedt D.C. (1994). Pheochromocytoma in 50 dogs. J. Vet. Intern. Med..

[30] Barrera J.S., Bernard F., Ehrhart E.J., Withrow S.J., Monnet E. (2013). Evaluation of risk factors for outcome associated with adrenal gland tumors with or without invasion of the caudal vena cava and treated via adrenalectomy in dogs: 86 cases (1993–2009). J. Am. Vet. Med. Assoc..

[31] Mayhew P.D., Culp W.T.N., Balsa I.M., Zwingenberger A.L. (2018). Phrenicoabdominal venotomy for tumor thrombectomy in dogs with adrenal neoplasia and suspected vena caval invasion. Vet. Surg..

[32] Mayhew P.D., Kirpensteijn J., Fransson B.A., Mayhew P.D. (2015). Laparoscopic adrenalectomy. Small Animal Laparoscopy and Thoracoscopy.

[33] Herrera M.A., Mehl M.L., Kass P.H., Pascoe P.J., Feldman E.C., Nelson R.W. (2008). Predictive factors and the effect of phenoxybenzamine on outcome in dogs undergoing adrenalectomy for pheochromocytoma. J. Vet. Intern. Med..

[34] Vasanjee S.C., Bubenik L.J., Hosgood G., Bauer R. (2006). Evaluation of hemorrhage, sample size, and collateral damage for five hepatic biopsy methods in dogs. Vet. Surg..

[35] McDevitt H.L., Mayhew P.D., Giuffrida M.A., Brown D.C., Culp W.T., Runge J.J. (2016). Short-term clinical outcome of laparoscopic liver biopsy in dogs: 106 cases (2003–2013). J. Am. Vet. Med. Assoc..

[36] Buote N.J., Kovak-McClaran J.R., Schold J.D. (2011). Conversion from diagnostic laparoscopy to laparotomy: Risk factors and occurrence. Vet. Surg..

[37] Petre S.L., McClaran J.K., Bergman P.J., Monette S. (2012). Safety and efficacy of laparoscopic hepatic biopsy in dogs: 80 cases (2004–2009). J. Am. Vet. Med. Assoc..

[38] Vaden S.L., Levine J.F., Lees G.E., Groman R.P., Grauer G.F., Forrester S.D. (2005). Renal biopsy: A retrospective study of methods and complications in 283 dogs and 65 cats. J. Vet. Intern. Med..

[39] Rawlings C.A., Diamond H., Howerth E.W., Neuwirth L., Canalis C. (2003). Diagnostic quality of percutaneous kidney biopsy specimens obtained with laparoscopy versus ultrasound guidance in dogs. J. Am. Vet. Med. Assoc..

[40] Crivellenti L.Z., Cianciolo R., Wittum T., Lees G.E., Adin C.A. (2018). Associations of patient characteristics, disease stage, and biopsy technique with the diagnostic quality of core needle renal biopsy specimens from dogs with suspected kidney disease. J. Am. Vet. Med. Assoc..

[41] Nowicki M., Rychlik A., Nieradka R., Kander M., Depta A., Chrzastowska M. (2010). Usefulness of laparoscopy guided renal biopsy in dogs. Pol. J. Vet. Sci..

[42] Al-Qudah H.S., Rodriguez A.R., Sexton W.J. (2007). Laparoscopic management of kidney cancer: Updated review. Cancer Control.

[43] Deane L.A., Clayman R.V. (2007). Laparoscopic nephrectomy for renal cell cancer: Radical and total. BJU Int..

[44] Marcovich R., Williams A.L., Seifman B.D., Wolf J.S. (2001). A canine model to assess the biochemical stress response to laparoscopic and open surgery. J. Endourol..

[45] Mayhew P.D., Mehler S.J., Mayhew K.N., Steffey M.A., Culp W.T. (2013). Experimental and clinical evaluation of transperitoneal laparoscopic ureteronephrectomy in dogs. Vet. Surg..

[46] Song J., Kim E., Mobley J., Vemana G., Tanagho Y., Vetter J., Bhayani S., Russo P., Fugita O., Yang S.S. (2014). Port site metastasis after surgery for renal cell carcinoma: Harbinger of future metastasis. J. Urol..

[47] Lopez D., Singh A., Wright T.F., Gartley C., Walker M. (2017). Single incision laparoscopic-assisted ovariohysterectomy for an ovarian tumor in a dog. Can. Vet. J..

[48] Hayes H.M., Pendergrass T.W. (1976). Canine testicular tumors: Epidemiologic features of 410 dogs. Int. J. Cancer.

[49] Runge J.J., Mayhew P.D., Case J.B., Singh A., Mayhew K.N., Culp W.T. (2014). Single-port laparoscopic cryptorchidectomy in dogs and cats: 25 cases (2009–2014). J. Am. Vet. Med. Assoc..

[50] Bleakley S., Duncan C.G., Monnet E. (2015). Thoracoscopic lung lobectomy for primary lung tumors in 13 dogs. Vet. Surg..

[51] Lansdowne J.L., Monnet E., Twedt D.C., Dernell W.S. (2005). Thoracoscopic lung lobectomy for treatment of lung tumors in dogs. Vet. Surg..

[52] Mayhew P.D., Hunt G.B., Steffey M.A., Culp W.T., Mayhew K.N., Fuller M., Johnson L.R., Pascoe P.J. (2013). Evaluation of short-term outcome after lung lobectomy for resection of primary lung tumors via video-assisted thoracoscopic surgery or open thoracotomy in medium-to large-breed dogs. J. Am. Vet. Med. Assoc..

[53] Monnet E., Fransson B.A., Mayhew P.D. (2015). Thoracoscopic Lung Biopsy and Lung Lobectomy. Small Animal Laparoscopy and Thoracoscopy.

[54] Mehlhaff C.J., Mooney S. (1985). Primary pulmonary neoplasia in the dog and cat. Vet. Clin. N. Am. Small Anim. Pract..

[55] Dhumeaux M.P., Haudiquet P.R. (2009). Primary pulmonary osteosarcoma treated by thoracoscopy-assisted lung resection in a dog. Can. Vet. J..

[56] Laksito M.A., Chambers B.A., Yates G.D. (2010). Thoracoscopic-assisted lung lobectomy in the dog: Report of two cases. Aust. Vet. J..

[57] Wormser C., Singhal S., Holt D.E., Runge J.J. (2014). Thoracoscopic-assisted pulmonary surgery for partial and complete lung lobectomy in dogs and cats: 11 cases (2008–2013). J. Am. Vet. Med. Assoc..

[58] Fourdrain A., De Dominicis F., Iquille J., Lafitte S., Merlusca G., Witte-Pfister A., Meynier J., Bagan P., Berna P. (2018). Intraoperative conversion during video-assisted thoracoscopy does not constitute a treatment failure. Eur. J. Cardio-Thorac. Surg..

[59] Hennon M.W., Kumar A., Devisetty H., D’Amico T., Demmy T.L., Groman A., Yendamuri S. (2019). Minimally invasive approaches do not compromise outcomes for pneumonectomy: A comparison using the National Cancer Database. J. Thorac. Oncol..

[60] Adamiak Z., Holak P., Piorek A. (2008). Thoracoscopic biopsy of lung tumors using a Roeder’s loop in dogs. Pol. J. Vet. Sci..

[61] Mayhew P.D., Culp W.T., Pascoe P.J., Arzi N.V. (2012). Use of the Ligasure vessel-sealing device for thoracoscopic peripheral lung biopsy in healthy dogs. Vet. Surg..

[62] MacIver M.A., Case J.B., Monnet E.L., Hunt G.B., Mayhew P.D., Oblak M.L., Runge J.J., Singh A., Smeak D.D., Steffey M.A. (2017). Video-assisted extirpation of cranial mediastinal masses in dogs: 18 cases (2009–2014). J. Am. Vet. Med. Assoc..

[63] Alwen S.G., Culp W.T., Szivek A., Mayhew P.D., Eckstrand C.D. (2015). Portal site metastasis after thoracoscopic resection of a cranial mediastinal mass in a dog. J. Am. Vet. Med. Assoc..

[64] Brisson B.A., Reggeti F., Bienzle D. (2006). Portal site metastasis of invasive mesothelioma after diagnostic thoracoscopy in a dog. J. Am. Vet. Med. Assoc..

[65] Ployart S., Libermann S., Doran I., Bomassi E., Monnet E. (2013). Thoracoscopic resection of right auricular masses in dogs: 9 cases (2003–2011). J. Am. Vet. Med. Assoc..

[66] Crumbaker D.M., Rooney M.B., Case J.B. (2010). Thoracoscopic subtotal pericardiectomy and right atrial mass resection in a dog. J. Am. Vet. Med. Assoc..

[67] Weisse C., Soares N., Beal M.W., Steffey M.A., Drobatz K.J., Henry C.J. (2005). Survival times in dogs with right atrial hemangiosarcoma treated by means of surgical resection with or without adjuvant chemotherapy: 23 cases (1986–2000). J. Am. Vet. Med. Assoc..

[68] Libermann S., Monnet E., Fransson B.A., Mayhew P.D. (2015). Right Auricular Mass Resection. Small Animal Laparoscopy and Thoracoscopy.

[69] Ehrhart N., Ehrhart E.J., Willis J., Sisson D., Constable P., Greenfield C., Manfra-Maretta S., Hintermeister J. (2002). Analysis of factors affecting survival in dogs with aortic body tumors. Vet. Surg..

[70] Dunning D., Monnet E., Orton E.C., Salman M.D. (1998). Analysis of prognostic indicators for dogs with pericardial effusion: 46 cases (1985–1996). J. Am. Vet. Med. Assoc..

[71] Case J.B., Maxwell M., Aman A., Monnet E.L. (2013). Outcome evaluation of a thoracoscopic pericardial window procedure or subtotal pericardectomy via thoracotomy for the treatment of pericardial effusion in dogs. J. Am. Vet. Med. Assoc..

[72] Atencia S., Doyle R.S., Whitley N.T. (2013). Thoracoscopic pericardial window for management of pericardial effusion in 15 dogs. J. Small Anim. Pract..

[73] Jackson J., Richter K.P., Launer D.P. (1999). Thoracoscopic partial pericardiectomy in 13 dogs. J. Vet. Intern. Med..

[74] Barbur L.A., Rawlings C.A., Radlinsky M.G. (2018). Epicardial exposure provided by a novel thoracoscopic pericardectomy technique compared to standard pericardial window. Vet. Surg..

[75] Dupre G.P., Corlouer J.P., Bouvy B. (2001). Thoracoscopic pericardectomy performed without pulmonary exclusion in 9 dogs. Vet. Surg..

[76] Fernandez J.A., Robles R., Acosta F., Sansano T., Parrilla P. (2004). Cardiovascular changes during drainage of pericardial effusion by thoracoscopy. Br. J. Anaesth..

[77] Linden D.S., Cole R., Tillson D.M., Boothe H.W., Matz B.M. (2018). Sentinel lymph node mapping of the canine anal sac using lymphoscintigraphy: A pilot study. Vet. Radiol. Ultrasound.

[78] Majeski S.A., Steffey M.A., Fuller M., Hunt G.B., Mayhew P.D., Pollard R.E. (2017). Indirect computed tomographic lymphography for iliosacral lymphatic mapping in a cohort of dogs with anal sac gland adenocarcinoma: Technique description. Vet. Radiol. Ultrasound.

[79] Liss M.A., Noguchi J., Lee H.J., Vera D.R., Kader A.K. (2015). Sentinel lymph node biopsy in bladder cancer: Systematic review and technology update. Indian J. Urol..

[80] Worley D.R. (2014). Incorporation of sentinel lymph node mapping in dogs with mast cell tumours: 20 consecutive procedures. Vet. Comp. Oncol..

[81] Tuohy J.L., Worley D.R. (2014). Pulmonary lymph node charting in normal dogs with blue dye and scintigraphic lymphatic mapping. Res. Vet. Sci..

[82] Lim H., Kim J., Li L., Lee A., Jeong J., Ko J., Lee S., Kweon O.K., Kim W.H. (2017). Bilateral medial iliac lymph node excision by a ventral laparoscopic approach: Technique description. J. Vet. Med. Sci..

[83] Steffey M.A., Daniel L., Mayhew P.D., Affolter V.K., Soares J.H., Fuller M.C. (2015). Laparoscopic extirpation of the medial iliac lymph nodes in normal dogs. Vet. Surg..

